# Sickle cell anaemia in vaso-occlusive crisis and acute fatty liver of pregnancy: a case report

**DOI:** 10.4314/gmj.v54i3.12

**Published:** 2020-09

**Authors:** Michael C Ezeanochie, Oghenefegor E Olokor, Ofure E Yamah

**Affiliations:** 1 Department of Obstetrics and Gynaecology, University of Benin Teaching Hospital, Benin city, Edo State, Nigeria

**Keywords:** Vaso-occlusive crises, acute fatty liver of pregnancy, hypoglycaemia, Sickle-cell anaemia

## Abstract

Acute fatty liver of pregnancy (AFLP) is an uncommon condition that manifests in the third trimester of pregnancy. Its association with vaso-occlusive crisis from Sickle Cell Anaemia is not common. Published data on the simultaneous occurrence of these two conditions is rare, hence this case report. A 32-year-old gravida 3 para 1+^1^ lady, with Sickle Cell Anaemia, had a vaso-occlusive crisis in association with AFLP at 32 weeks' gestation, and the outcome of her management was successful.

AFLP is a rare late-gestational event affecting about 1 in 10,000 to 15,000 pregnancies. The exact aetiology is not known. Profound hypoglycaemia and jaundice with elevated serum transaminases are recognized features of AFLP. These features may also be seen in haemoglobinopathies. The simultaneous occurrence of AFLP and Sickle Cell Anaemia may result in overlap of symptoms and delay in diagnosis and treatment. Therefore, maintaining a high index of suspicion is key. The cornerstone for treatment remains prompt delivery and supportive care.

AFLP can coexist with Sickle cell crises. It is important that care providers, especially in populations with high burden of Sickle Cell Anaemia, consider this as a differential diagnosis, especially when the jaundice is associated with profound or recurring episodes of hypoglycaemia. Prompt diagnosis and delivery in a multidisciplinary approach is important to avoid adverse maternal and foetal outcomes.

**Funding:**

No funding sources

## Introduction

Acute fatty liver of pregnancy (AFLP) is a rare late-gestational event affecting about 1 in 10,000 to 1 in 15,000 pregnancies.^1^ The exact aetiology is unknown, however long chain 3-hydroxyacyl coenzyme A dehydrogenase (LCHAD) enzyme defect and abnormal fatty acid oxidation have been implicated.^2^ Patients typically present with nausea, vomiting, and abdominal pain, which are non-specific symptoms. This tends to cause a delay in diagnosis, hence the high morbidity and mortality in patients with AFLP.^1^

Profound hypoglycaemia and jaundice are features of AFLP that could also be present in other conditions such as the haemoglobinopathies.^3^ Sickle cell anaemia is one of the inheritable haemoglobinopathies, commonly characterized by features of chronic haemolytic anaemia and vaso-occlusive crisis.^4^ It is associated with multi-organ dysfunction and can affect the liver as well; this may manifest with hypoglycaemia.^3^,^5^

Although the aetiologic trigger of AFLP is not well known, and the possibility exists that liver dysfunction as a result of Sickle Cell Anaemia may be a trigger for AFLP in pregnancy. In the face of sickle cell anaemia, AFLP can pose a diagnostic challenge. The simultaneous occurrence of both diseases may result in an overlap of symptoms such as jaundice. Therefore, a high index of suspicion is necessary. Prompt delivery and supportive care remain the modality for treatment before disseminated intravascular coagulopathy, a major complication of AFLP develops.^2^ This report is a pregnant woman with Sickle Cell Anaemia in vaso-occlusive crisis associated with AFLP at 32 weeks gestation. Published data on the simultaneous occurrence of these two conditions is extremely rare, hence this case report.

## Case Report

A 32-year-old Gravida 3 para 1+^1^ with one previous caesarean section 18years ago, was referred to us at 32 weeks gestation, as a case of Sickle Cell Anaemia (HbSS) in vaso-occlusive crisis for specialist care. She had presented at the referral centre with a history of generalized body pains, passage of dark coloured urine and fever of a week duration. There was no vomiting; there was associated orthopnoea and easy fatiguability. She had no complaints of labour pains, bleeding per vaginam, liquor drainage or reduced foetal movement. There was no history of drug use other than her routine haematinics. Her last crisis was a year before presentation. Her steady state Packed Cell Volume (PCV) was 25%, but her PCV at presentation in the referring centre was 16%. She was transfused with four units of blood and referred to us for specialist care as her clinical condition continued to deteriorate.

When seen at our hospital, she was conscious but drowsy, acutely ill with a temperature of 37.5°C. She was pale and icteric. She was dehydrated and had bilateral pitting pedal oedema. Her respiratory rate was 33 cycles per minute, and there were fine crepitations in the lung bases with a partial pressure of oxygen (room air) at 88%. Her pulse was 116 beats per minute, and blood pressure was 128/75 mmHg. The first and second heart sounds were heard and there were no added sounds. Her abdomen was uniformly enlarged, the symphysio-fundal height was 32cm, which was compatible with her gestational age. Foetal heart tones were regular at 140 beats per minute. The liver and spleen were not palpable, and the kidneys were not ballotable. She was admitted as a case of sickle cell anaemia with haemolytic crises.

Her PCV was 24%. There was leucocytosis of 23.5x10^3^/ul. Platelet count was 193,000/ul, Urea and Creatinine were 33mmol/l and 194umol/l respectively, which were both elevated. Screening for HIV 1 and 2 infections was negative. There was no protein in the urine. An obstetric bedside Ultrasound scan wan normal for the gestational age. The random blood sugar at admission was 0.67mmol/l. Malaria parasite test and urine culture were negative.

She was managed by a multidisciplinary team of obstetricians, intensivists, haematologist, and nurses. She was nursed in cardiac position and placed on oxygen via nasal prongs. Hypoglycaemia was corrected with infusion of 50% dextrose, and she was placed on maintenance infusion of 10% dextrose. She was on normal saline alternating with dextrose infusions (3 litres every 24 hours), she was catheterized, and there was strict input and output monitoring. She was placed on 0.5mg of oral Folic acid daily, 120mg of intravenous artesunate stat then 60mg in 4, 24 and 48 hours. In addition, she received intravenous ceftriaxone 1 gm daily, oral paracetamol 1gm 8 hourly and frusemide 40mg 8hrly. She was transfused with two units of packed red cells. Subsequently her packed cell volume ranged between 23% and 25%.

She had recurring episodes of hypoglycaemia despite correction with intravenous dextrose infusions. Her clinical state began to deteriorate on the seventh day with worsening leucocytosis of 32.1 × 10^3^ cells/uL, Urea and Creatinine of 52mmol/l and 362umol/l respectively. At this point, Acute fatty liver of pregnancy was considered. The liver function test was deranged with elevated transaminases (ALT- 34 and AST- 31) u/l respectively and a mildly elevated serum bilirubin of 19.3umol/l. Screening for viral markers of Hepatitis B and C were negative. The clotting profile, prothrombin and activated partial thromboplastin time were normal.

The multidisciplinary team, including the neonatologists, reviewed her case, and the decision was to terminate the pregnancy at this point, and she consented. She had a Caesarean section with the delivery of a live male 1.9kg neonate with APGAR scores of 8 and 9 in the first and fifth minutes of life respectively. The estimated blood loss was 500ml. The baby was admitted to the neonatal unit.

Postoperatively; she was managed in the intensive care unit. Her PCV was 23% and she received one unit of packed red cell. She had three episodes of hypoglycaemia within 48 hours of delivery that were successfully corrected. Otherwise, her clinical condition progressively improved after delivery. The liver function tests and serum electrolytes, urea and creatinine levels were normal by the 5th post postoperative day. Her clinical state continued to improve; she was transferred from the ICU to the maternity ward by the 7^th^ day and discharged home with her baby on the 10^th^ day post-partum. At the 6th week post-delivery, she was seen with her baby at the postnatal clinic and both of them were in satisfactory condition. She was counselled on contraceptive choices and referred to the Haematology clinic for further followup.

## Discussion

AFLP is an uncommon condition characterized by deposition of fat in the liver cells, causing destruction of hepatocytes.^1^ This has been attributed to long chain 3-hydroxyacyl coenzyme A dehydrogenase (LCHAD) enzyme defect and abnormal fatty acid oxidation.^1^ It is usually a diagnosis of exclusion.^1^,^2^ Its association with sickle cell anaemia is rare and is not established in the literature.

The clinical features of AFLP are usually non-specific and may include, abdominal pain, nausea and vomiting.^2^ Also, profound hypoglycaemia and jaundice have been strongly associated with AFLP.^1^,^2^,^4^ The exact aetiology is not known, but identified risk factors include, multiple gestation, male foetuses and first pregnancies.^6^ Our patient presented with generalized body pains, fever, jaundice and hypoglycaemia.

These symptoms were consistent with sickle cell crises from malaria in pregnancy. However, her poor response to treatment of malaria and vaso-occlusive crisis in the referring hospital and recurring episodes of hypoglycaemia in our hospital led to the suspicion of AFLP. The deranged liver function tests with elevated transaminases are in keeping with intrahepatic jaundice, which is consistent with a diagnosis of AFLP.

AFLP commonly manifests in the third trimester.^2^ There is currently no standardized method for diagnosis.^6^ A constellation of clinical findings and investigations are used to make a diagnosis. It is a progressive disease that tends to regress following delivery.^4^,^6^ Our patient was counselled for preterm delivery to avoid deterioration in her condition and that of the foetus. The marked improvement in her clinical condition and the results of laboratory investigations after delivery reinforced the diagnosis of AFLP.

There is a high risk of maternal morbidity and mortality in AFLP from haemorrhage, renal failure, and sepsis. The need for immediate delivery, often preterm, increases the risk of perinatal morbidity and mortality from prematurity. These risks are further heightened with concurrent comorbidities like sickle cell anaemia. Multidisciplinary care is important in the management of AFLP and Sickle cell anaemia in pregnancy. This has been shown to reduce maternal morbidity and mortality markedly.^7^,^8^,^9^ The simultaneous occurrence of AFLP with Sickle cell anaemia makes multidisciplinary care imperative. Our patient was provided care in a multidisciplinary setting which may have contributed to the favourable pregnancy outcome.

## Conclusion

We conclude that, although rare, AFLP can coexist with sickle cell crises. There is a need for additional research to find out if sickle cell anaemia increases the risk of AFLP. It is important that care providers, especially in populations with high burden of sickle cell anaemia, consider this as a differential diagnosis especially when the jaundice is associated with profound or recurring episodes of hypoglycaemia. Prompt diagnosis and delivery in a multidisciplinary team setting is important to avoid adverse maternal and foetal outcome.
